# Meta‐analysis of the diagnostic value of exosomal miR‐21 as a biomarker for the prediction of cancer

**DOI:** 10.1002/jcla.23956

**Published:** 2021-09-07

**Authors:** Fanglan Liu, Haifei Mao, Shiquan Chai, Haifeng Mao

**Affiliations:** ^1^ Department of Preclinical Medical Jiangxi Medical College Shangrao China; ^2^ Department of Anesthesiology Shangrao People’s Hospital Shangrao China; ^3^ Department of Anesthesiology Taizhou First People’s Hospital Taizhou China

**Keywords:** cancer, exosomes, meta‐analysis, microRNA‐21

## Abstract

**Background:**

Early diagnosis of cancer is still the most effective method to increase survival and therapeutically effective patient management. Accumulating studies had exploited exosomes as an indicator for the diagnosis and prognosis of cancer. In addition to exosomes, exosome‐derived miRs are widely investigated as a novel biomarker for diagnosis in cancer patients. The aim of this study was to clarify the diagnostic value of ex‐miR‐21 in cancer.

**Methods:**

Databases were searched for eligible studies up to June, 2021. Studies included in this meta‐analysis were reviewed and selected independently by two authors. The data of sensitivity, specificity, diagnostic odds ratio (DOR), and summary receiver operating characteristic curves (SROC) of exosomal miR‐21 as a diagnostic biomarker were extracted and calculated. Quality assessment was conducted by using the QUADAS‐2 tool.

**Results:**

A total of 26 studies were included in the systematic analysis and meta‐analysis. The pooled results of sensitivity, specificity, PLR/NLR, DOR, and area under the curve were 76% (95%CI, 0.70–0.81), 82% (0.77–0.87), 4.3 (3.1–6.0), 0.29 (0.22–0.38), 15 (8–26), and 0.86 (0.83–0.89), respectively. Sensitivity analysis and Deeks' funnel plot indicated that results remained unchanged and had no publication bias. For the subgroup analysis, it was showed that ex‐miR‐21 had a superior diagnostic accuracy on identifying PC.

**Conclusion:**

Exosomal microRNA‐21 can serve as an effective and widely used diagnostic biomarker for cancer, especially in PC. The using field of exosomes and exosome‐derived miR can further extend the prognosis and therapeutic management. Standardized isolation of exosomes and miRNA‐21 should be developed.

## Graphical abstract

The role of miR‐21 as a potential serum/plasma biomarker has been determined while they are still unperforming in cancer diangosis. Currently, the application of miR‐21 from exosomes has been demonstrated and its diagnostic accuracy should be identified as well.

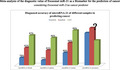

## 1 INTRODUCTION

Cancer and its high incidence have become a serious threat to human health and bring a great challenge to treatment planning and research progress.^1^ Timely diagnosis and therapeutic monitoring remain one of the major challenges during the treatment of disease. Lacking a reliable detection method, many cases of cancer were misdiagnosed or delayed diagnosed and missed the best treatment time eventually.^2^ The five‐year survival rate was poor in several types of cancer as the disease is hardly detectable in its early stage and is often being found out until the late stages with limited treatment options.

Accumulating studies have demonstrated the positive correlation between exosomes and the biological process of cancer, such as tumor growth, metastasis, and chemoresistance.^3^, ^4^, ^5^ Applications of exosome‐shuttled proteins and nucleic acids in cancer diagnosis and prognosis have also caught attention in the detection of several types of cancers.^6^, ^7^ Given its high stability in the extracellular environment, less interference to serum and detectable in various body liquids (plasma, serum, urine, CSF, etc.), exosomes are advantaged as a novel biomarker for tumor diagnosis.

Exosomal miRNAs, small noncoding RNAs of approximately 18–25 nucleotides length, are one of the study subjects with the significant expression level of RNAs and are suggested as diagnostic and prognostic indicators for various types of cancer.^8^, ^9^ Among those microRNAs, exosomal mir‐21 is the only significantly overexpressed miRNA in a wide range of solid cancer. Current studies on the diagnosed accuracy of ex‐mir‐21disperse in a single type of cancer, such as lung,^10^ breast,^11^ and ovarian cancers,^12^ merely discussed the overall application of ex‐mir‐21 in different types of cancer. In this study, we perform a comprehensive analysis of the diagnostic performance of ex‐mir‐21 in cancer diagnosis.

## 2 MATERIALS AND METHODS

### 2.1 Literature search and search strategy

Eligible studies were identified in electronic databases that are recommended by the handbook and by cross‐checking the reference lists of relevant papers up to March 2021. The Cochrane Library, Embase via OVID, PubMed, ScienceDirect, and Web of Science databases were systematically searched using search terms: (“exosomes” OR “exosomal” OR “exocrine”), (“microRNA‐21” OR “microR‐21” OR “mir‐21”), (“biomarker” OR “marker, biological”), and (“neo‐plasms” OR “cancer” OR “carcinoma” OR “tumor”) as well as their abbreviations, text words, and subject terms without time limitation and language restriction. The search strategies were adjusted to different databases, and the search strategy of PubMed was presented as an example: (((exosomal [Title/Abstract] OR exosome [Title/Abstract] OR exosomes[Title/Abstract]) AND (microRNA‐21[Title/Abstract] OR microR‐21[Title/Abstract] OR miR‐21[Title/Abstract])) AND (biomarker[Title/Abstract] OR marker, biological[Title/Abstract])) AND (tumor[Title/Abstract] OR cancer[Title/Abstract] OR carcinoma[Title/Abstract] OR adenocarcinoma[Title/Abstract]).

### 2.2 Inclusion and exclusion criteria

Two investigators (FL and HM) independently extracted data from the eligible papers complying with the inclusion and exclusion criteria. Disagreements were subsequently reviewed and resolved through discussion. Studies were included in the meta‐analysis if they met the following inclusion: (1) studies investigating the role of ex‐mir‐21 in the diagnosis of cancer; (2) study patients with any type of carcinoma should be confirmed by the gold standard; (3) studies reported the detailed clinical data that can be used to calculate diagnostic accuracy data, including and the number of true‐positive (TP), false‐positive (FP), true‐negative (TN), and false‐negative (FN) cases, 2×2 diagnostic table; (4) articles written in English. Animal experiments were excluded, and only original articles were considered. Other publications, including letters, reviews, case reports, or editorial articles, were excluded.

### 2.3 Quality assessment

The quality and potential bias of the studies were assessed using the QUADAS‐2 tool in the Review Manager. The risk of bias for each study was ranked as “low,” “unclear,” and “high.” The same two investigators assessed the study quality independently, and discrepancies were resolved by discussion. The risk of bias level was ranked according to four different areas: (i) patient selection, (ii) index test, (iii) reference standard, and (iv) flow of patients through the study and timing of the index tests and reference standard (flow and timing).

### 2.4 Statistical analysis

TP, TN, FP, and FN rates in the included studies were the primary data to assess the sensitivity, specificity, positive likelihood ratio (PLR), negative likelihood ratio (NLR), diagnostic odds ratios (DORs), and SROC. The diagnostic performance of ex‐mir‐21 was determined by calculating those values with 95% CIs. The DOR was used to reflect the relationship between diagnostic tests and disease where higher numbers would indicate improved performance in diagnosing patients with/without cancer. The DOR summarized the diagnostic accuracy of the index test as a single number that describes how many times higher the odds were of obtaining a test positive result in a diseased, rather than a non‐diseased person. PLR/NLR was a ratio of the probability that a test result is correct to the probability that the test is incorrect. The larger the ratio of PLR, the greater is the probability of being a true positive when the result is positive. The smaller the ratio of NLR, the greater is the probability of being a true negative when the result is negative. Summary receiver operating characteristic (SROC) curves were generated to estimate the effect of sensitivity and specificity based on TP and FP rates. The area under the curve (AUC) of the SROC was also calculated to assess the performance of ex‐mir‐21 in diagnosing cancer. A prediction interval (the 95% prediction contour line in Figure 3) was used to consider the potential effect of the biomarker in the cancer diagnosis when it is applied within an individual setting.^13^

The heterogeneity between studies was assessed using the χ^2^ test and the inconsistency index (I^2^). An I^2^ > 50% with *p* < 0.05 from the χ^2^ test is indicative of significant heterogeneity. In this case, a random‐effects model was chosen to pool the data of sensitivity, specificity, and AUC. Otherwise, a fixed‐effects model was used. The threshold effect was considered a possible cause of heterogeneity in diagnostic accuracy analysis. Spearman correlation was used to analyze the logit of sensitivity and the logit of (1‐specificity) and to verify the existence of the threshold effect. A strong positive correlation (correlation > 0.6) between sensitivity and (1‐specificity), with *p* < 0.05 was considered to indicate a statistically significant threshold effect. If a certain variance could affect the heterogeneity and overall diagnostic effect, sensitivity analysis was performed by omitting one study at a time to examine the stability of the pooled results. Subgroup analysis was conducted to determine if a certain variance could affect heterogeneity. Several groups were conducted for subgroup analyses, including ethnicity (Caucasian‐based, Asian‐based), cancer types (digestive cancer, breast cancer, lung cancer, other types of cancer), sample source (plasma‐based, serum‐based, other sources of exosomes).

Deeks' funnel plot and an asymmetry test were used to assess publication bias, and a *p*‐value < 0.5 was considered having publication bias.

Software Stata (version 15.1) and Review Manager (version 5.1) were used to analyze the statistic. Quality assessment of the included studies was conducted using RevMan version 5.3 (Nordic Cochrane Centre; Cochrane Collaboration).

## 3 RESULTS

### 3.1 Study evaluation

**FIGURE 1:**
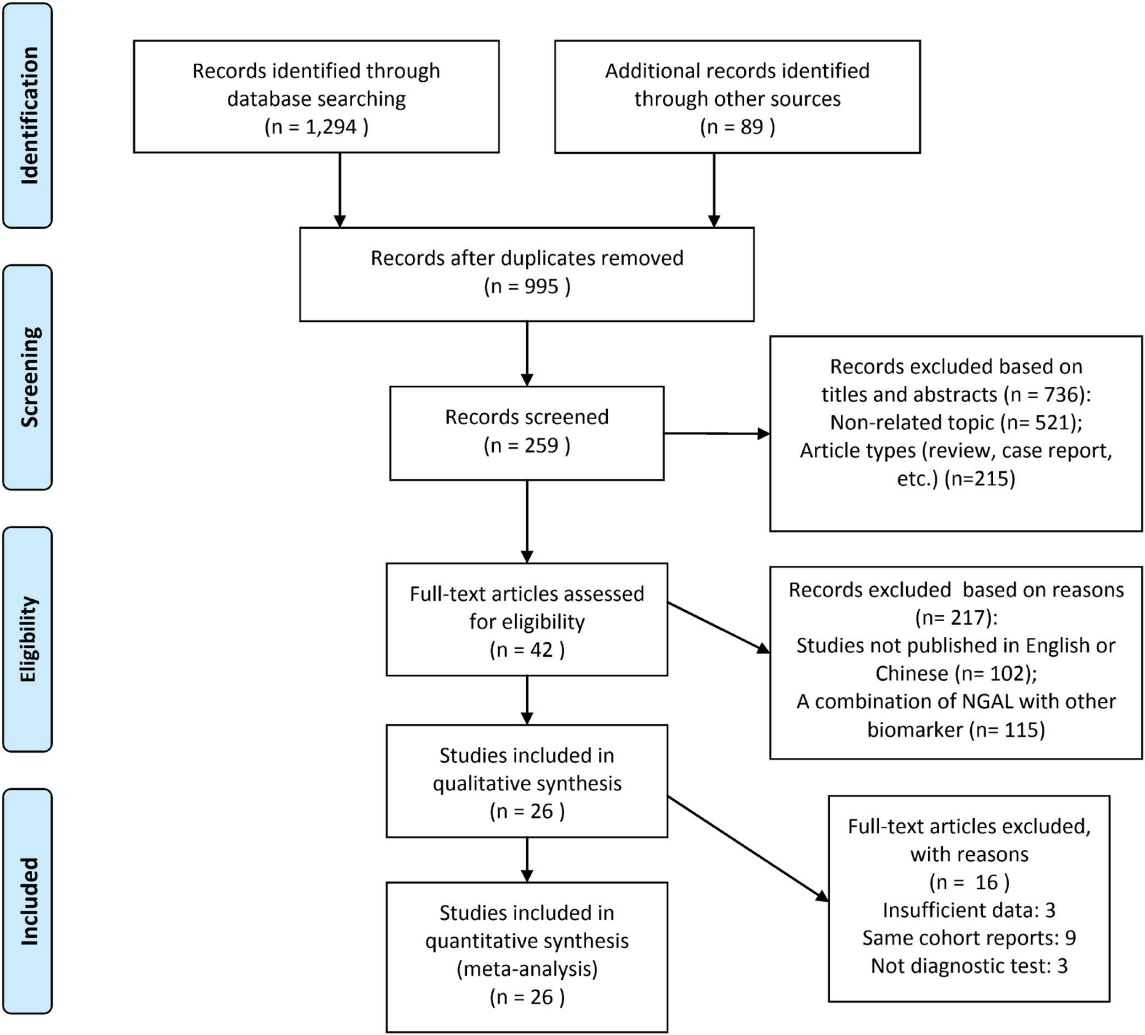
Flowchart of literature search and study selection

In total, 803 studies were identified in the search of multiple databases and cross‐checking for reference lists. After removing duplicates, 309 studies contained and went through a screening process based on titles and abstracts. One hundred fifty‐three studies were identified for full‐text review, and eighty‐six of them were excluded because of nondiagnostic study, review articles, or diagnostic accuracy data not included or calculable in the publication. Finally, 26 studies met the inclusion criteria and were included in the meta‐analysis and review.^10^, ^11^, ^12^, ^14^, ^15^, ^16^, ^17^, ^18^, ^19^, ^20^, ^21^, ^22^, ^23^, ^24^, ^25^, ^26^, ^27^, ^28^, ^29^, ^30^, ^31^, ^32^, ^33^, ^34^, ^35^, ^36^ A detailed flow diagram of the study selection is shown in Figure 1.

### 3.2 Study characteristics

Table 1 summarizes the characteristics of the 26 included studies in our meta‐analysis. There were 30 trials in the 26 articles with the publication years ranged from 2008 to 2020. The investigated cancer types were divided into four groups: digestive cancer (*n* = 13), breast cancer (*n* = 2), lung cancer (*n* = 3), and other types of cancer (*n* = 11), including colon cancer (CC), ovarian cancer (OC), hepatocellular carcinoma (HCC), glioblastoma, renal cell carcinoma (RCC), intraductal papillary mucinous neoplasm (IPMN), and lymphoma. Sample sources are consisted of plasma (*n* = 6), serum (*n* = 13), pancreatic juice (*n* = 1), CLF exosome (*n* = 1), peritoneal lavage fluid exosome (PLF) (*n* = 1), portal vein blood (*n* = 1), and peripheral blood (*n* = 1).

**TABLE 1:** Basic characteristics

Study	Publication year	Country	Sample size	Source	Cut‐off	Cancer	TP	FN	FP	TN	Refs.
Akers et al.	2013	The United States	27	CSF‐EV	0.25	Glioma	11	0	2	14	14
Ando et al.	2019	Japan	48	Urine	0.413	BC	16	5	6	21	11
Butz et al.	2015	Canada	46	Urine	−6.9	RCC	20	4	8	14	15
Goto et al.	2018	Japan	51	Serum	median miR−21 value	IPMN	22	4	7	18	16
			31			PC(early)	6	4	3	18	
			45			PC(advanced)	20	4	3	18	
Hernández‐Walias et al.	2020	Spain	111	Plasma	6.171	Lymphoma	25	21	12	53	17
Jin et al.	2019	China	43	Serum	The Youden index	CRC	11	14	0	18	18
Kawamura et al.	2018	Japan	55	Portal vein blood	1.18	PC	24	6	9	16	19
			55	Peripheral blood		PC	18	8	15	14	
Lai et al.	2017	The United States	35	Plasma	1.38	PC	29	0	0	6	20
Liu et al.	2014	China	70	CLF exosome	NR	Glioma	40	0	5	25	21
Liu et al.	2020	The United States	79	Serum	NR	LC	53	2	11	13	10
			79	Serum		LC	29	3	35	12	
Matsuzaki et al.	2017	Japan	60	Urine	The Youden index	UC	27	1	9	23	22
Melo et al.	2014	The United States	19	Serum	NR	BC	9	0	2	8	23
Nakamura et al.	2019	Japan	35	Pancreatic Juice	The Youden index	PDAC	22	1	5	7	24
Ogata‐Kawata et al.	2014	Japan	99	Serum	1.08	CC	54	2	34	9	25
Pan et al.	2018	Germany	135	Plasma	NR	OC	65	5	41	24	12
Que et al.	2013	China	49	Serum	7.693	PC	21	5	1	22	26
Soeda et al.	2019	Japan	129	Plasma	0.93	GC	45	13	28	43	27
Tanaka et al.	2013	Japan	85	Serum	Median miR−21 value	ESCC	28	6	16	35	28
Taylor et al.	2008	The United States	40	Serum	NR	CRC	30	0	0	10	29
Tokuhisa et al.	2015	Japan	18	PLF exosome	NR	HCC	8	2	1	7	30
Tsukamoto et al.	2017	Japan	326	Plasma	Median miR−21 value	CRC	78	72	72	104	31
Uratani et al.	2016	Japan	73	Serum	The Youden index	CRC	18	9	8	38	32
Wang et al.	2014	China	43	Serum	5 fold	CRC	9	12	4	18	33
Wang et al.	2020	China	100	Plasma	NR	HCC	41	4	9	46	35
Wang J et al.	2014	China	101	Serum	0.043	Laryngeal squamous cell carcinoma	36	9	16	40	34
Yang et al.	2020	China	99	Serum	NR	LC	61	7	14	17	36

Abbreviations: BC, breast cancer; CC, colon cancer; CRC, colorectal cancer; CSF, cerebrospinal fluid; ESCC, esophageal squamous cell carcinoma; FN, false negative; FP, false positive; GC, gastric cancer; GC, gastric cancer; HCC, hepatocellular carcinoma; IPMN, intraductal papillary mucinous neoplasm; LC, lung cancer; PC, Pancreatic cancer; PDAC, pancreatic ductal adenocarcinoma; PSF, pancreatic cyst fluid; RCC, renal cell carcinoma; TN, true negative; TP, true positive.

### 3.3 Quality assessment

**FIGURE 2:**
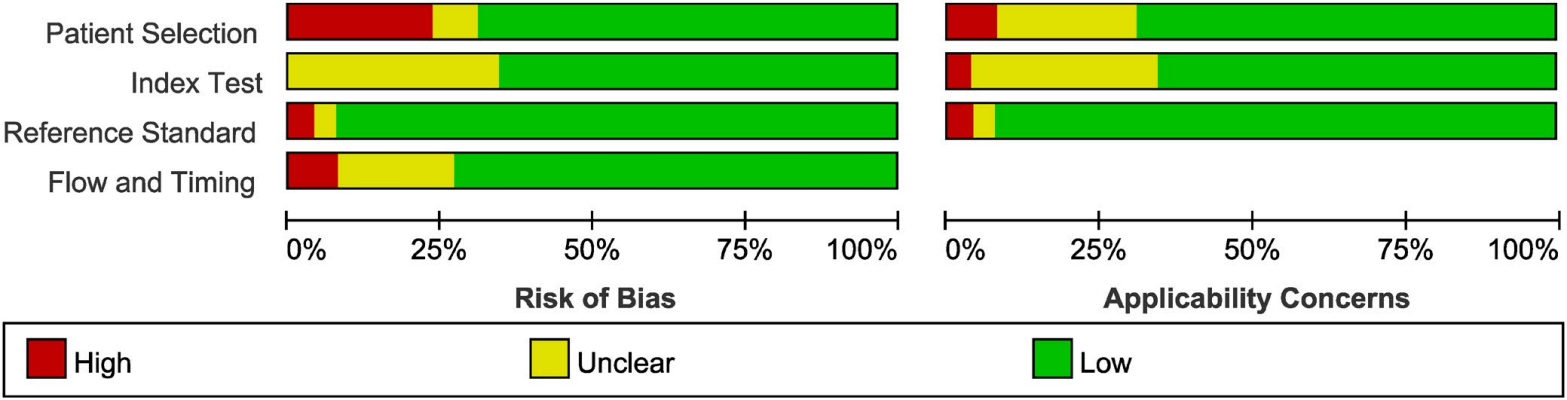
Results of quality assessment (high risk of bias: red, unknown: yellow, low risk: green)

Regarding the quality evaluation of the included studies, a majority of included studies had a low risk of bias. The risk of bias in the patient selection domain was considered having a relatively higher amount of bias in five of twenty‐seven included studies. These studies did not clarify if their studies avoid inappropriate exclusions. Several studies that did not mention its interpretation way of blindness were assessed as unclear or high risk of bias in the “index test” and “reference standard” category. Our ratings of the risk of bias and applicability concerns in each study are displayed in Figure 2.

**FIGURE 3:**
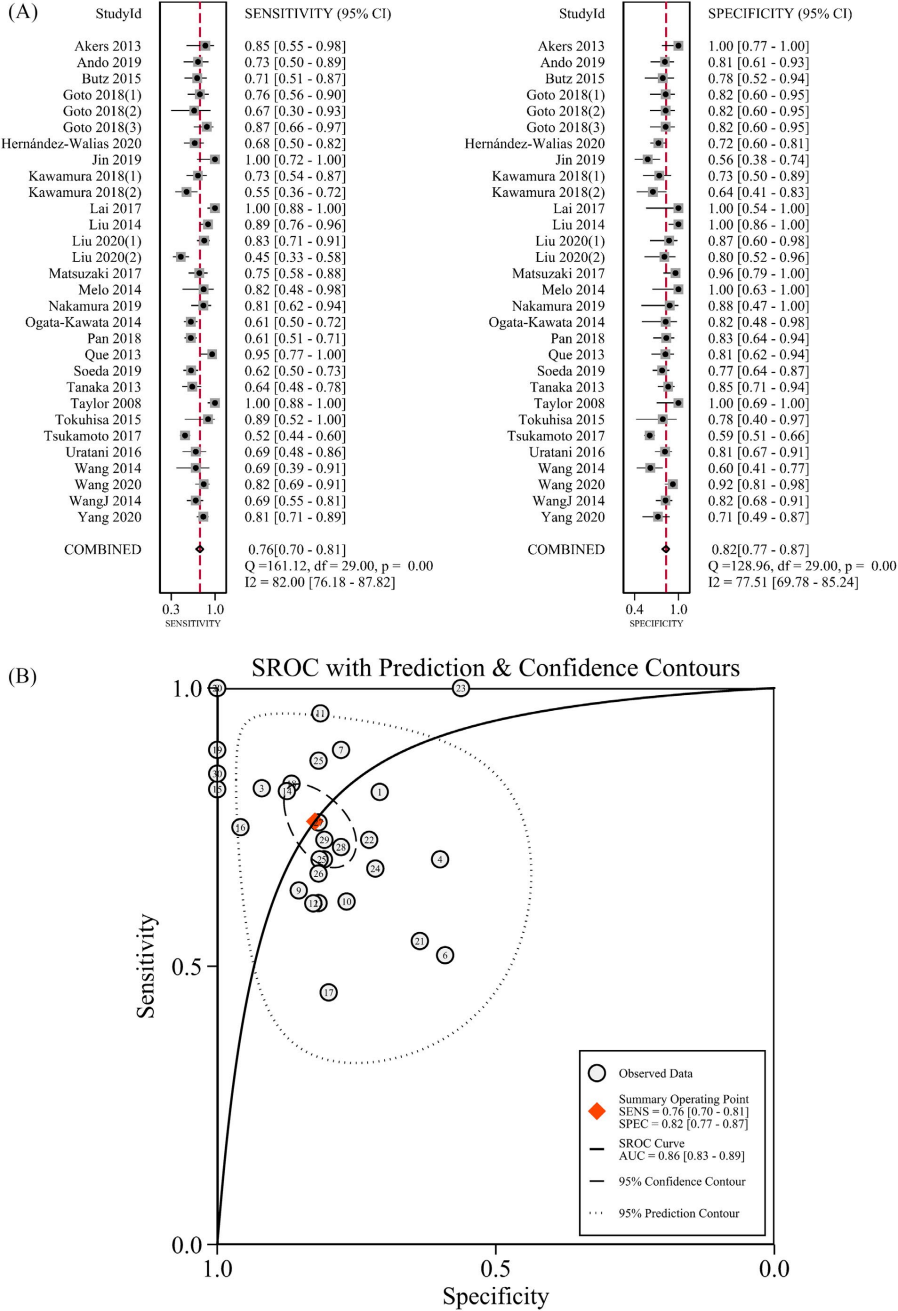
Diagnostic accuracy of exosomal miRNA‐21 in differentiating between malignant and benign tumors. Pooled sensitivity and specificity of exosomal miRNA‐21 (A); summary receiver operating characteristic curve of exosomal miRNA‐21 (B)

### 3.4 The output results of this meta‐analysis

The pooled sensitivity and specificity of the ex‐mir‐21 diagnostic accuracy were calculated by a random‐effects model. The overall sensitivity and specificity of ex‐mir‐21 for diagnosing cancer were 0.76 (95%CI, 0.70–0.81) and 0.82 (0.77–0.87), respectively (Figure 3). Predictive values (positive or negative) are obtained from estimates of sensitivity and specificity by creating an illustrative 2x2 table and computing predictive values directly. The positive predictive values remained relatively high (>75%) in 23 of 30 studies. PLR and NLR was 4.34 (3.14–5.99) and 0.29 (0.22–0.38) (Figure S1), respectively. The DOR of the meta‐analysis was 15 (8–26; Figure S2). The area under the curve was 0.86 (95%CI, 0.83–0.89; Figure 3). The area under the curve represents diagnostic accuracy, and the AUC of 0.86 indicating a relatively high diagnostic performance that the use of exosomal‐miR‐21 as a diagnostic biomarker of cancer is effective and feasible. A threshold effect (Spearman correlation coefficient: 0.83, *p* = 0.7) did not exist in the studies.

Outlier detection was conducted for the investigation of heterogeneity. Most appropriate is meta‐analysis with low risk of bias, although it also suggested that the source of heterogeneity may come from the studies of Jin et al.,^18^ Lai et al.,^20^ Matsuzaki et al.,^22^ Pan et al.,^12^ Que et al.,^26^ Taylor et al.,^29^ and Yang et al.^36^ (Figure S3). After excluding outliers (the study of Taylor et al. and Lai et al.), the overall sensitivity and specificity of ex‐mir‐21 diagnostic capability were 0.73 (0.67–0.78) and 0.81 (0.76–0.85) (Table 2), respectively. The AUC was 0.84 (0.80–0.87); omitting the study of Taylor et al. and Lai et al. decreased the heterogeneity to 0% (data not shown). The reason for heterogeneity may be the perfect sensitivity and specificity (100%) by a small amount of included participants.

**TABLE 2:** Summary estimates of diagnostic performance of miR‐21 for cancer detection

Analysis	Sensitivity (95% CI)	Specificity (95% CI)	PLR (95% CI)	NLR (95% CI)	DOR (95% CI)	AUC (95% CI)
Ethnicity						
Caucasian‐based	0.84 (0.65, 0.94)	0.91 (0.73, 0.98)	9.7 (2.6, 36.6)	0.17 (0.06, 0.46)	56 (6, 498)	0.95 (0.92, 0.96)
Asian‐based	0.73 (0.68, 0.79)	0.80 (0.74, 0.85)	3.7 (2.7, 4.9)	0.33 (0.26, 0.42)	11 (7, 18)	0.83 (0.8, 0.86)
Cancer types						
Digestive system	0.77 (0.68, 0.84)	0.78 (0.71, 0.84)	3.5 (2.4, 5.1)	0.3 (0.2, 0.45)	12 (6, 25)	0.84 (0.81, 0.87)
Pancreatic cancer	0.85 (0.71, 0.93)	0.84 (0.69, 0.92)	5.2 (2.5, 11)	0.17 (0.08, 0.39)	30 (7, 124)	0.91 (0.88, 0.93)
PDAC	0.76 (0.68, 0.84)	0.74 (0.61, 0.85)	2.7 (1.23, 5.91)	0.32 (0.14, 0.74)	11.75 (2.14, 64.65)	0.85 (0.74, 0.98)
Gastric cancer	0.65 (0.53, 0.75)	0.77 (0.65, 0.87)	2.82 (1.76, 4.51)	0.37 (0.13, 1.1)	7.52 (2, 28.26)	N.A.
Breast cancer	0.76 (0.58, 0.89)	0.85 (0.69, 0.95)	4.24 (1.92, 9.35)	0.30 (0.16, 0.54)	14.71 (4.20, 51.58)	N.A.
NSCLC	0.70 (0.64, 0.77)	0.78 (0.64, 0.88)	3.00 (1.82, 4.94)	0.34 (0.14, 0.79)	9.73 (3.11, 30.46)	0.85 (0.71, 0.98)
Other types	0.71 (0.65, 0.78)	0.87 (0.79, 0.92)	5.4 (3.1, 9.1)	0.33 (0.25, 0.43)	16 (7, 35)	0.85 (0.81, 0.87)
Sample types						
Plasma‐based	0.79 (0.69, 0.86)	0.79 (0.73, 0.84)	3.8 (2.9, 5)	0.27 (0.18, 0.4)	14 (8, 20)	0.85 (0.81, 0.88)
Serum‐based	0.74 (0.54, 0.87)	0.84 (0.62, 0.95)	4.8 (1.4, 16.1)	0.31 (0.14, 0.7)	15 (2, 115)	0.86 (0.83, 0.89)
Other types	0.75 (0.66, 0.82)	0.86 (0.76, 0.92)	5.4 (2.9, 10.2)	0.29 (0.20, 0.43)	18 (7, 50)	0.87 (0.84, 0.89)
Overall	0.76 (0.70–0.81)	0.82 (0.77–0.87)	4.34 (3.14 – 5.99)	0.29 (0.22 – 0.38)	15 (8–26)	0.86 (0.83–0.89)
Two outliers excluded	0.73 (0.67–0.78)	0.81 (0.76–0.85)	3.8 (2.9–5.0)	0.34 (0.27–0.42)	11 (7–18)	0.84 (0.80–0.87)

Abbreviations: AUC, area under the curve; DOR, diagnostic odds ratios; N.A., not applicable; NLR, negative likelihood ratio; NSCLC, non‐small‐cell lung cancer; PDAC, pancreatic ductal adenocarcinoma; PLR, positive likelihood ratio.

To account for the potential sources of heterogeneity, sensitivity and subgroup analyses were also conducted. The heterogeneity was still unchanged, and no significant difference was detected in the sensitivity analysis by omitting each of the included studies (data not shown). During the subgroup analysis, the pooled sensitivity, specificity, PLR, NLR, DOR, and AUC for each subgroup were calculated and are presented in Table 2. There was an obvious difference between the pooled data in ethnicity analysis that the diagnostic accuracy of ex‐mir‐21 was superior in studies with Caucasian‐based compared with Asian‐based (sen: 84% vs 73%, spc: 91% vs 80%, AUC: 95% vs 83%), implicating that the ethnicity may be a potential factor impacting on heterogeneity and the diagnostic accuracy. The cancer types divided into four cancer types revealed a less effective performance of ex‐mir‐21 in diagnosing NSCLC (sen: 70%, spc: 78%) and other types of cancer (sen: 71%, spc: 87%). According to a subdivided analysis, our results suggested that ex‐mir‐21 has an excellent diagnostic performance in the diagnosis of PC (sen:85%, spc:84%, AUC: 91%). On the other side, there was no significant difference between the pooled data in sample sources; the diagnostic accuracy was slightly better in the group of serum‐based studies revealing that ex‐mir‐21 showed the versatility to detect cancer from various bodily fluids of humans. According to Deeks’ funnel plot asymmetry test, no publication bias was detected among the studies (*p* = 0.703, Figure S4).

## 4 DISCUSSION

Exosomes have increasingly come to the front as important sources of reliable biomarkers for cancer diagnosis and prognosis.^37^, ^38^ Accumulating evidence has suggested that miRNAs, IncRNAs, and proteins containing exosomes isolated from body fluid are significantly different between cancer patients and healthy.^39^, ^40^ It is suggested that those ex‐miRNAs play an important role in tumor immune escape that the information contained in them can reprogram the functions of immunologically active factors and immune target cells.^41^ We gathered complete literature and pooled the diagnostic values of ex‐mir‐21. The overall estimate of the meta‐analysis demonstrated that ex‐mir‐21 has a sensitivity and specificity of 76% and 82% in the early detection of cancer, respectively. An AUC of 0.86 further indicated a high accuracy in diagnosing and differential diagnosis of tumors. However, a lot is still unknown or in a dispute about ex‐mir‐21. Thus, several subgroup analyses were also conducted in an attempt to reveal its true capability.

Subgroup analysis based on cancer types showed that ex‐miR‐21 had a superior diagnostic accuracy in identifying cancer in the digestive system, especially PC (AUC: 0.91, I^2^ = 1.28%). Pancreatic juice cytology (PJC) has been used to diagnose PDAC in case of gastric wall implantation or dissemination of tumor cells; however, its sensitivity was relatively lower than that of ex‐mir‐21 (40%–60%), although combining positive results of the ex‐miR‐21 level to PJC increased the sensitivity to 93% and the specificity to 88%.^24^ These findings raise the possibility of exosomal‐miR usage in the development of specific biomarkers for PDAC diagnosis and extension to other digestive cancer. Furthermore, the usage of ex‐mir‐21 can be expanded to the detection of cancer recurrence, prognosis, and chemoresistance.^16^, ^18^, ^31^ Tsukamoto et al. investigated ex‐miR‐21 as a marker of CRC prognosis and reported that plasma ex‐miR‐21 is a useful biomarker for poor prognosis in CRC patients at TNM stage II, III, or IV.^31^ Even though its accuracy in diagnosing lung cancer was comparatively lower than other systems (sen: 70%, spc: 78%, AUC: 0.85), Liu et al. have developed a sensitive biochip to detect exosomal miRNAs in human sera and achieved higher detection sensitivity and specificity.^10^ In the study of Yang et al., the combination of ex‐mir‐21 with the Let‐7a ratio held a promising accuracy in the differentiation between NSCLC and benign pulmonary diseases.^36^ Even so, the sample size of the pooled study was limited, and the performance of ex‐mir‐21 in the early diagnosis of NSCLS or lung cancer should be further validated in larger cohorts. The estimate on the diagnosis of breast cancer has a similar situation that a small number of studies were included in the meta‐analysis, and further investigation should be considered. Apart from cancer types, we also assessed their diagnostic accuracy from different fluids. The results showed that ex‐mir‐21 from serum had the highest specificity but a low sensitivity, implicating that ex‐mir‐21 isolated from serum is more effective in the identification of healthy but would misclassify patients with a true malignant tumor. Many studies have identified mir‐21 in the exosome from different bodily fluids, such as CSF (cerebrospinal fluid) and pancreatic and other tumor juice, and its diagnostic ability in cancer detection.^10^, ^19^, ^25^ In fact, exosome‐derived miR has been found to remain stable at −208°C for 5 years and to be resistant to freeze‐thaw cycles.^42^ However, due to the limited amount of included studies, only the studies with exosomes derived from plasma and serum were possible to conduct a subgroup analysis; and studies using other types of samples were classified together. These results suggested that ex‐mir‐21 is an effective biomarker for cancer diagnosis and can be applied to different cancer types.

Circulating mir‐21 can also distinguish malignant tumors with an AUC of 0.84, the sensitivity of 75%, and specificity of 79.9% in gastric cancer, pancreatic cancer, esophageal cancer, etc.^43^, ^44^ However, Wang et al. reported a negative opinion toward circulating mir‐21 as its inferior diagnostic likelihood ratios were not enough to rule out cancer.^45^ Extracellular miR‐21 was investigated by Qu et al.^46^ in a two‐phase study with a meta‐analysis and an experiment on the secretory mechanisms of extracellular mir‐21 in glioma cells. They concluded a high accuracy of extracellular mir‐21 in the diagnosis of cancers, specifically in the diagnosis of brain cancers (AUC 0.94). In this regard, the efficacy of extracellular mir‐21 might be similar to ex‐mir‐21. Additionally, for both of them, lipid membrane coverage protects miRNA from RNases degradation, making them more stable than circulating mir‐21. However, the boundary of extracellular vesicles and exosomes is vague as exosomes were described as extracellular vesicles 30 years ago. The confusing use of extracellular vesicles may exist between studies currently.^47^ The current study has suggested that miRNA from cancer cells are more concentrated in exosomes that the concentration of ex‐miR‐21 was 213, 10 times greater than that in the cells, which means ex‐miR‐21 can be a potentially excellent candidate of a biomarker for cancer conditions as miRNAs are easily lost in the body fluids due to nucleases exposure.^48^ During the exploration and quantification of miRNA containing exosomes, ex‐mir‐21 is the only overexpressed sample in a wide range of cancer patients, indicating that it can provide autocrine and paracrine signals to the surrounding microenvironment and influence the growth of malignancy.^8^, ^49^ Moreover, ex‐mir‐21 was able to represent the status of tumor progression as the high expression of ex‐mir‐21 is associated with low survival and poor outcomes in HCC and CRC,^50^ which is because tumor‐derived ex‐mir‐21 converted the hepatic stellate cells (HSCs) to cancer‐associated fibroblasts (CAFs), which further secrete exosomal‐mir‐21 to promote HCC progression.^50^ Therefore, more and more attention has been paid to the role of miR‐21 in prevention and therapeutic strategies.

The study of exosomes is an active area of research. The outstanding performance of ex‐mir‐21 is significantly higher than that of CA19‐9 and CEA, whose diagnostic abilities were specific for certain types of cancers. Its high stability in circulation and microenvironment and reproducible detection also advantage its further application in the clinical setting. However, to utilize exosomal miRNAs as a comprehensive diagnosis biomarker, standardized isolation of exosomes and miRNA should be developed due to the complex biosystem and the expensive laboratory equipment, making it more practical for application. Then, using field of exosomes can extend the therapeutic management: a predictor of tumor response to treatment and a vehicle for medical therapy. miRNA expression affects signaling pathway components during chemotherapy, radiotherapy, and targeted therapies. Exosomes can load miRNAs, targeting and combining fundamental genetic molecules in the pathways mediating chemotherapy, radiotherapy, and targeted therapy.

### 4.1 Limitation

Several points should be a concern before clinical application. First of all, the cutoff value of exomiRNA‐21 was a consideration in a diagnostic analysis as a consensus has not been reached currently even though no threshold existed. Second, there may be a selection bias in the subgroup analysis of ethnicity and cancer types. The patient population was classified into Asian‐based and Caucasian‐based, without including African population. Also, the diagnostic accuracy was inferior in the subgroup analysis of breast cancer and lung cancer because a small number of studies were included (BC:2, LC:3). Therefore, further investigations are encouraged.

## 5 CONCLUSION

A favorable and preferred choice of ex‐mir‐21 as an effective biomarker in diagnosing cancer is recommended and shows strong potential in clinical settings. For a better diagnosis of each cancer type, we should investigate the clinical use of exosomal mir‐21 in specific cancers with a larger amount of enrollment. Prospectively, exploring the potential prognostic role of exosomes will contribute to the management of the therapeutic strategies.

## CONFLICT OF INTEREST

The authors declare no conflicts of interest.

## Supporting information

Fig S1‐S4
