# Supplementary material for: Meta‐analysis of the diagnostic value of exosomal miR‐21 as a biomarker for the prediction of cancer
Source: J Clin Lab Anal. 2021 Sep 7;35(10):e23956. doi: 10.1002/jcla.23956 (PMC8529139; doi:10.1002/jcla.23956)

Content

[1. Supplementary Figure 1 Diagnostic accuracy of exosomal miRNA-21 in the meta-analysis: (left) positive likelihood ratio, (right) negative likelihood ratio. 2](#_Toc77249181)

[2. Supplementary Figure 2 Diagnostic accuracy of exosomal miRNA-21 in the meta-analysis: diagnostic odds ratio. 3](#_Toc77249182)

[3. Supplementary Figure 3 Sensitivity analysis of the overall pooled studies. 4](#_Toc77249183)

[4. Supplementary Figure 4 Diagnostic accuracy of exosomal miRNA-21 in the meta-analysis: Deeks'funnel plot. 5](#_Toc77249184)

## Supplementary Figure 1 Diagnostic accuracy of exosomal miRNA-21 in the meta-analysis: (left) positive likelihood ratio, (right) negative likelihood ratio.


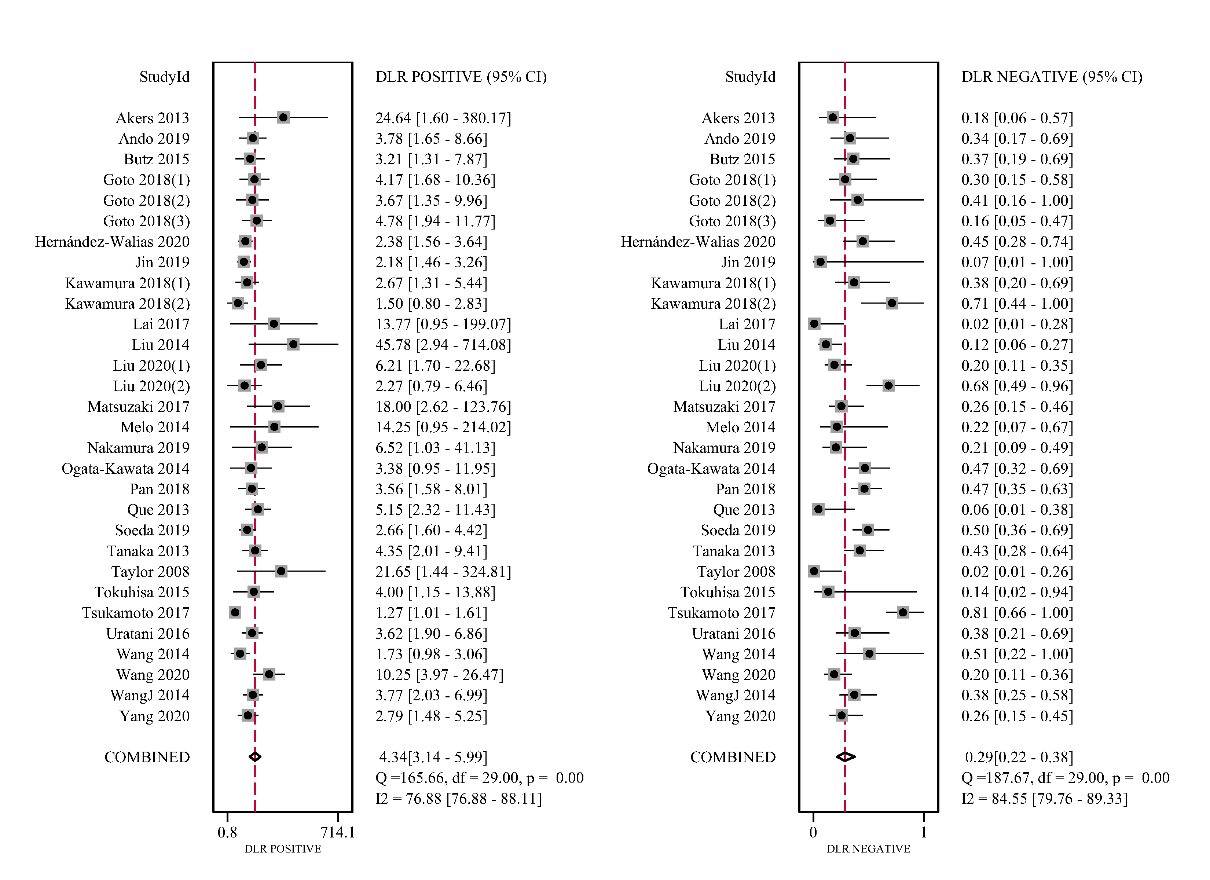


## Supplementary Figure 2 Diagnostic accuracy of exosomal miRNA-21 in the meta-analysis: diagnostic odds ratio.


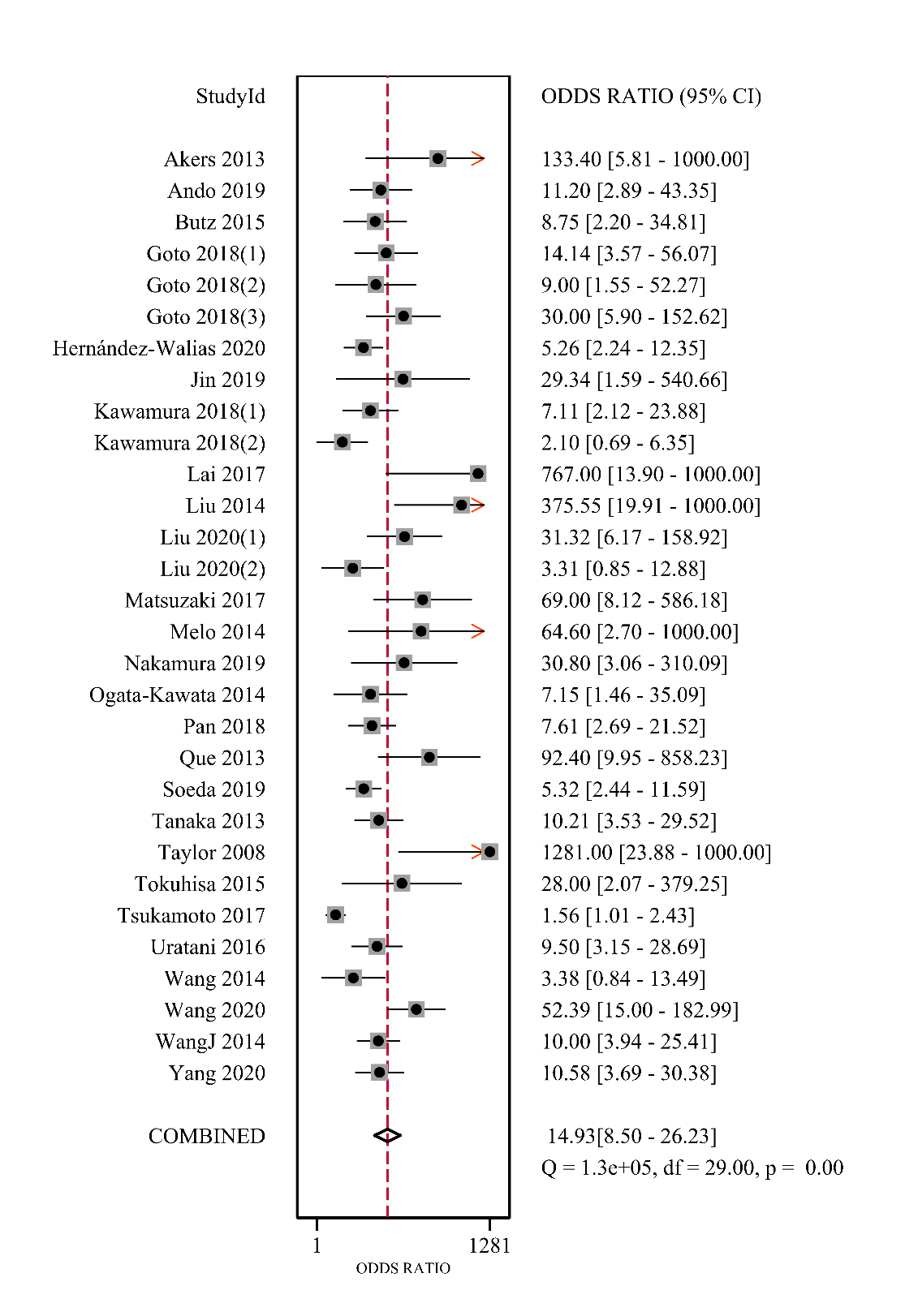


## Supplementary Figure 3 Sensitivity analysis of the overall pooled studies*


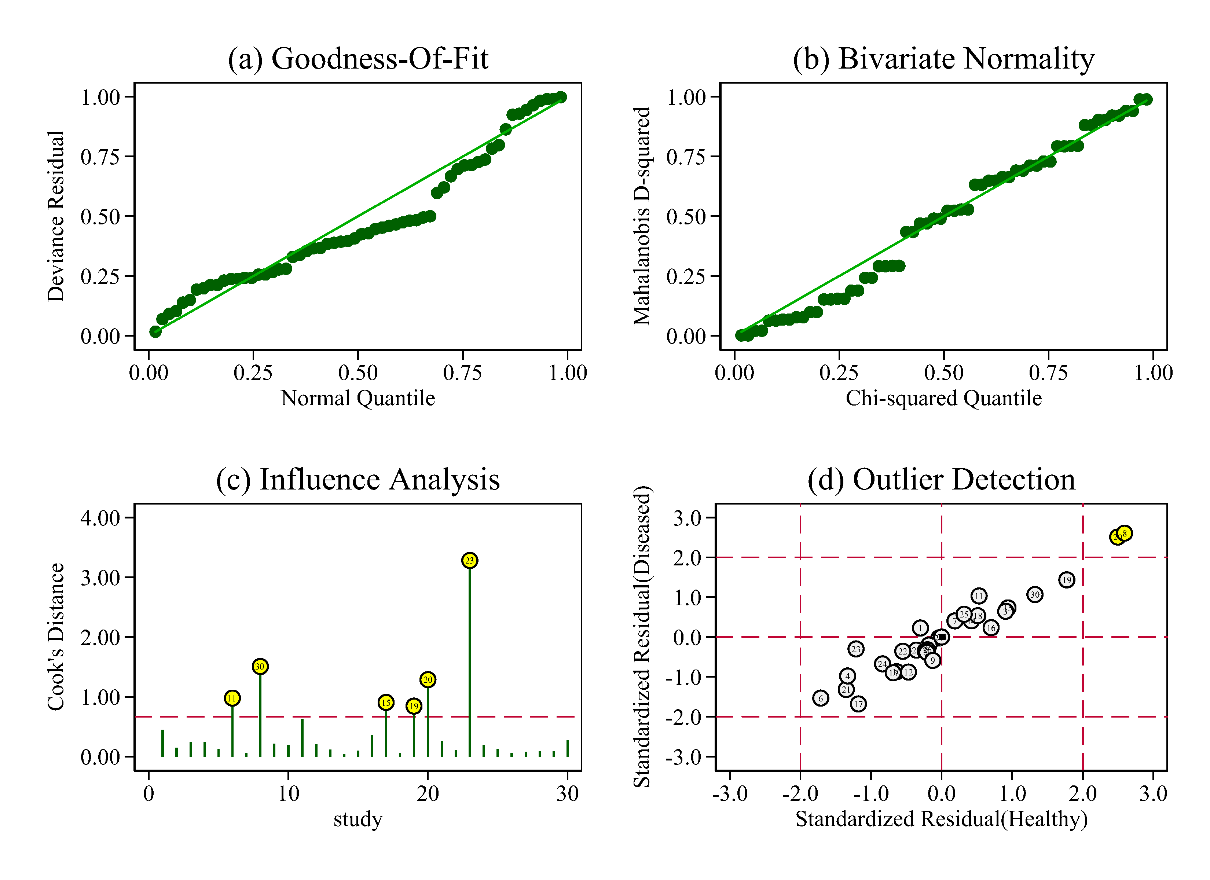


*Those numbers are the included studies listed in alphabetical order (A-Z) by the first letter of the first author. (the study of Jin et al, Lai et al , Matsuzaki et al, Pan et al, Que et al, Taylor et al , Yang et al)

## Supplementary Figure 4 Diagnostic accuracy of exosomal miRNA-21 in the meta-analysis: Deeks'funnel plot.


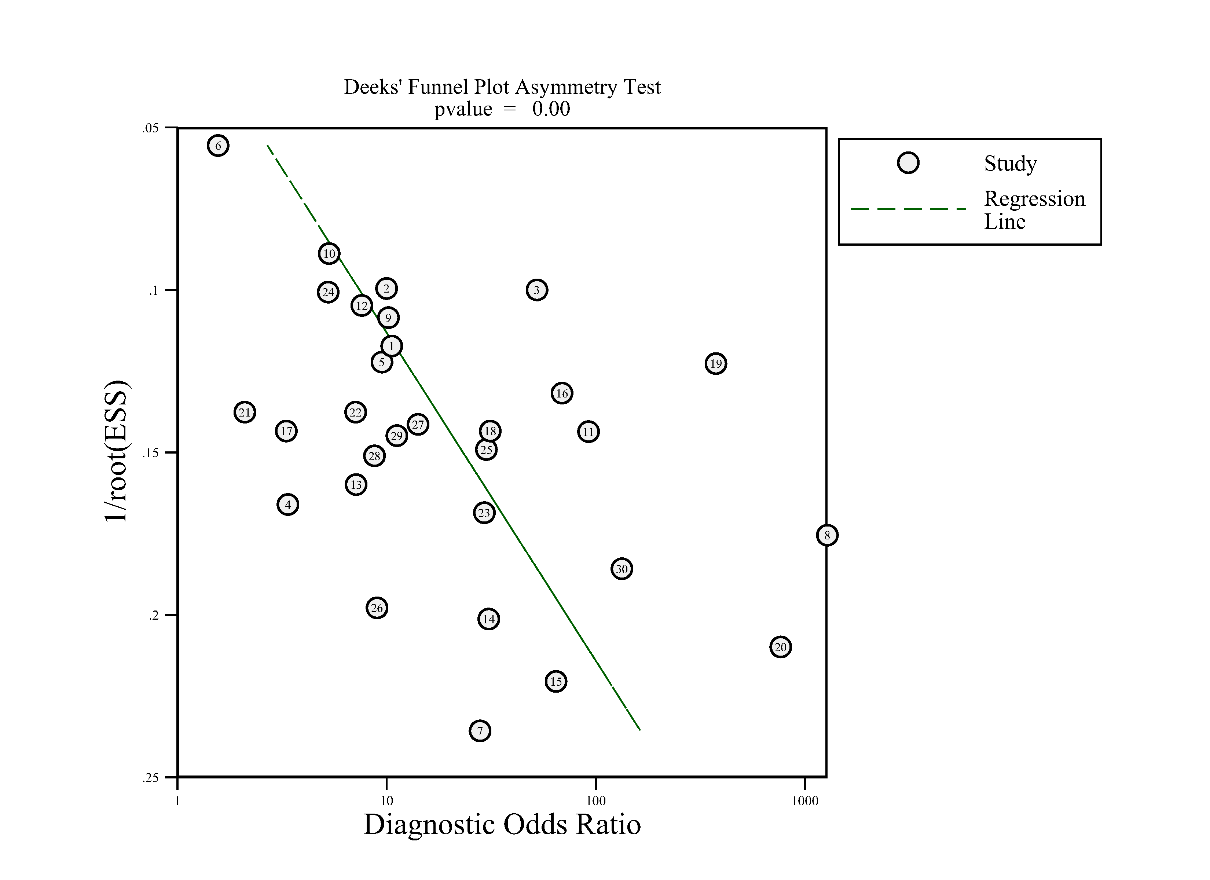

Supplement: Supplementary file 1 — Fig S1‐S4 [file JCLA-35-e23956-s001.docx]
